# Upper Limb Deep Vein Thrombosis: A Case Report of an Increasingly Common Condition

**Published:** 2018-04

**Authors:** Alexander W.Y. Chen, Kaveh Oraii Yazdani, Luciano Candilio

**Affiliations:** 1 *Cardiology Department, East Surrey Hospital, Redhill, UK. *; 2 *Cardiovascular Intervention Department, Tehran Heart Center, Tehran University of Medical Sciences, Tehran, Iran.*

**Keywords:** *Upper extremity deep vein thrombosis*, *Thromboembolism*, *Axillary vein*, *Subclavian vein*

## Abstract

Upper limb deep vein thrombosis (DVT) is a less common phenomenon than lower limb DVT. Repeated trauma secondary to sport- or job-related arm movements and positions has been recognized as the predisposing factor for upper limb DVT. We describe a 38-year-old male computer programmer admitted with swelling and pain in his left upper limb. Venous duplex ultrasound confirmed the presence of axillary vein thrombosis. Coagulation studies for secondary thrombosis were unremarkable. The patient was treated with full anticoagulation using low molecular weight heparin and warfarin. On subsequent follow-up at 3 months, the patient was symptom free and duplex sonography showed no evidence of thrombosis.

## Introduction

Lower limb deep vein thrombosis (DVT) is a common and well-described condition which has recently grown in public awareness. The risk factors for lower limb DVT arise from the underlying components of Virchow’s triad: venous stasis, hypercoagulability, and injury to the intima of veins.

Upper limb thrombosis, involving the axillary or subclavian vein, is a less common phenomenon.^1^ This condition is subject to the same risk factors as the formation of lower limb DVT. Another way of assessing the risk factors for upper limb DVT is by considering endogenous (e.g., thrombophilia and pregnancy) and exogenous (e.g., external compression of the vein by the cervical rib or a solid tumor) causes, although it may occur spontaneously.^2^

The relatively fixed position of the axillo-subclavian vein in the thoracic inlet/outlet predisposes it to repeated trauma with arm movements, leading to the compression of the vein in the costo-clavicular space. The axillo-subclavian compression between the clavicle and the first rib may also be exaggerated with frequent strenuous arm movements (e.g., tennis and body building) or when the upper extremity is in particular positions such as the rigid military style of sitting with the back straight and the shoulders placed posteriorly and inferiorly (e.g., sitting at a computer desk).^3^

We report a case of spontaneous axillary vein thrombosis and predict that the incidence of this condition will increase.

## Case Report

A 38-year-old left-hand dominant man woke up with acute pain and swelling in his left upper limb. He was a computer programmer and, additionally, used to work as a disc jockey in a local nightclub every weekend. He had no intrinsic risk factors for thromboembolic disease but spent prolonged periods of the day with his upper limbs in a relatively stationary position whilst using a computer keyboard.

**Figure 1:**
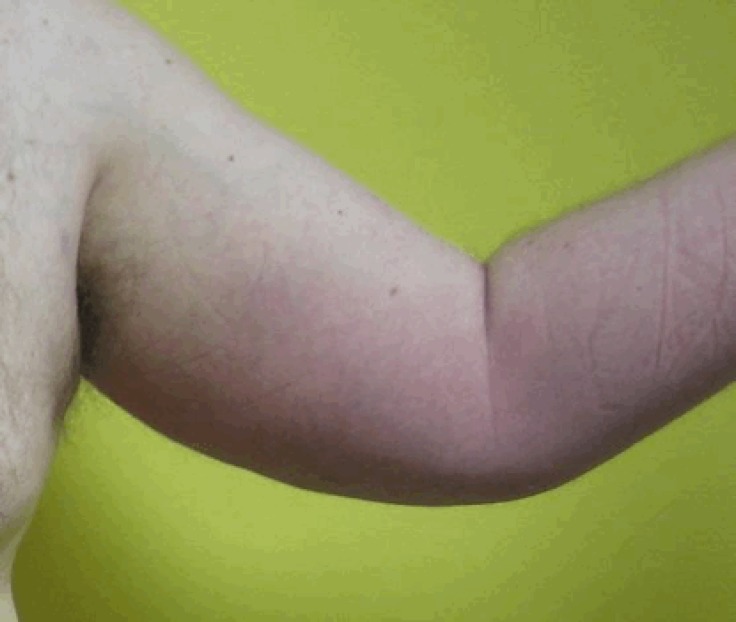
Patient’s left arm appearance at presentation

Physical examination revealed erythema, heat, swelling, and tenderness localized to the ulna border of his left arm (Figure 1). There was no systemic feature of illness.

Laboratory tests including routine full blood count, renal and liver profiles, inflammatory markers, thrombophilia test, and viral screen were unremarkable, with the exception of elevated D-dimer.

Venous duplex ultrasound of his left arm demonstrated compressible radial, ulnar, and brachial veins with decreased compressibility in the left axillary vein and confirmed the presence of thrombosis extending throughout the length of the vessel. Although there was no obvious symptom or sign of pulmonary embolism, we decided to proceed with computed tomography pulmonary angiography (CTPA), given the patient’s Wells score of 3 (proven DVT), which corresponds to an intermediate risk category for pulmonary embolism (score ≥2 and ≤6).^4^ CTPA showed no evidence of pulmonary embolism. 

We treated the patient with full anticoagulation using subcutaneous low molecular weight heparin and oral warfarin, and his signs and symptoms gradually resolved over the next few days. On subsequent follow-up at 3 months, he remained well and asymptomatic and repeated venous Doppler ultrasound revealed complete resolution of the left axillary thrombosis. Following the guidelines from the American College of Chest Physicians, which recommend anticoagulation for a minimum of 3 months for all patients identified with uncomplicated primary upper extremity DVT, we discontinued his warfarin.^5^

## Discussion

Thrombosis of the subclavian vein was first described in 1875 by Sir James Paget,^6^ who named it “gouty phlebitis”. However, he incorrectly attributed this syndrome to vasospasm rather than to thrombosis. In 1884, von Schrötter^7^ independently described this condition and postulated that it resulted from occlusive thrombosis of the subclavian or axillary veins. In recognition of these physicians, Hughes^8^ coined the term “Paget–Schrötter syndrome” in 1949 to describe the syndrome of spontaneous primary thrombosis of the axillary or subclavian vein.

The estimated incidence of DVT from all causes is 131.5 per 100 000 person-years. Nonetheless, this may be an underestimation because a number of DVTs remain asymptomatic and therefore undiagnosed.^9^ Prior to 1967, thrombosis of the axillary or subclavian vein was estimated to account for 1% to 2% of all DVTs. Since then, its incidence has risen due to increasing usage of central venous cannulation (CVC) and it is now estimated that upper limb DVT makes up 4% to 10% of all episodes of DVTs, with an approximate annual incidence of 3.6 per 100 000 persons.^10^ Thrombosis is thought to occur in the dominant arm in 80% of cases.^3^

A thorough history taking and an appropriate physical examination are usually sufficient to suggest this diagnosis.^11^ This may then be confirmed with a venogram or venous ultrasound scan. Differentiating between primary and secondary upper limb venous thrombosis is crucial in view of their different natural histories. Primary thrombosis accounts for 20% to 50% of all upper limb DVT s.^10^^, ^^12^ It includes real idiopathic cases and Paget–Schrötter syndrome, which is attributed to repetitive effort. 

Major risk factors for secondary DVT are CVC, cancer, and upper extremity surgery.^13^ Approximately, between 30% and 40% of all upper limb DVTs are cancer-related and 70% of secondary cases are related to CVC.^14^ In a study, 38.5% of the patients who had a peripherally inserted central catheter line developed upper limb DVTs.^15^

As is the case for lower limb DVT, the diagnosis of upper limb DVT is crucial considering its potentially life-threatening complications such as pulmonary thromboembolism.^16^

We predict that the incidence of upper limb DVT as a whole will increase. The incidence of secondary upper limb DVT will increase due to more frequent usage of subclavian vein catheters, especially in patients with cancers, who are already at higher risk of developing this condition. The use of permanent pacemakers is also increasing, which will further add to the incidence of this disease. With our population’s increasingly sedentary lifestyle and the increasing employment opportunities in the information-technology industry with repetitive workload on the upper limb in an almost fixed position, we predict that the future will yield many more similar cases to our case report.

## Conclusion

Upper limb thrombosis is a less known and rarer condition than lower limb thrombosis and has been particularly associated with exogenous factors, including sports and occupation. Nevertheless, diagnosis of upper limb DVT is similarly crucial, and recognition and treatment of potential complications such as pulmonary embolism are vital. 

## Notes:

***This paper should be cited as: ***Chen AW, Oraii Yazdani K, Candilio L. Upper Limb Deep Vein Thrombosis: A Case Report of an Increasingly Common Condition. J Teh Univ Heart Ctr 2018;13(2):73-75.
